# Isoflavone-enriched soybean leaves (Glycine max) restore loss of dermal collagen fibers induced by ovariectomy in the Sprague Dawley rats

**DOI:** 10.1186/s42826-024-00189-4

**Published:** 2024-02-14

**Authors:** Dae Young Yoo, Cheng-liang Xie, Joo Yeon Jeong, Ki Hun Park, Sang Soo Kang, Dong Hoon Lee

**Affiliations:** 1 Department of Anatomy and Convergence Medical Science, Institute of Medical Science, College of Medicine, Gyeongsang National University, Jinju, 52727 Republic of Korea; 2 Department of Anatomy and Cell Biology, College of Veterinary Medicine, and Research Institute for Veterinary Science, Seoul National University, Seoul, 08826 Republic of Korea; 3 College of Ecology, Lishui University, Zhejiang, 323000 China; 4 Division of Applied Life Science (BK21 Plus), IALS, Gyeongsang National University, Gyeongsang National University, Jinju, 52828 Republic of Korea

**Keywords:** Isoflavone, Soybean leaves, Collagen, Skin, Menopause, Ovariectomy

## Abstract

Phytoestrogens, such as isoflavones, are known for their capacity to simulate various physiological impacts of estrogen in the human body. Our research evaluated the effects of isoflavone-enriched soybean leaves (IESL) on collagen fiber loss prompted by ovariectomy in Sprague Dawley (SD) rats, thereby simulating menopausal changes in women. IESL, bolstered with an increased concentration of isoflavones through a metabolite farming process, contained a significantly higher amount of isoflavones than regular soybean leaves. Our results indicate that the administration of IESL can counteract the decrease in relative optical density and dermal thickness of collagen fibers caused by ovariectomy in SD rats, with more pronounced effects observed at higher isoflavone dosages. These outcomes suggest that soybean leaves rich in isoflavones may hold potential benefits in combating collagen degradation and skin aging symptoms related to menopause. Further research is needed to fully understand the exact molecular pathways at play and the potential clinical relevance of these findings.

## Background

Menopause is a normal phenomenon that occurs in women, and hormonal changes after menopause cause many physiological symptoms in the women's bodies [1]. When women undergo hormonal changes, they experience mental stress, such as depression and anxiety, but they also suffer from many physical symptoms, such as osteoporosis, metabolic disorders, and skin aging [2]. The skin, the outermost defense line of the body, is considerably associated with environmental factors, but it is also significantly affected by aging and menopausal hormonal changes [3]. Estrogen receptors (ERs) are expressed in multiple tissues, including the skin, and ERα and β are linked to collagen biosynthesis [4]. ER-dependent reactions are closely related to skin health and have a distinct effect on skin protection through anti-inflammatory action [5]. Agonists for ERα and ERβ showed anti-inflammatory effects during the recovery phase of the skin [6]. Specifically, clinical studies that utilize topical estrogens and topical isoflavones, soy-derived compounds that interact with estrogen receptors, are discussed [7].

Phytoestrogens, which include isoflavonoids, flavonoids, stilbenes, and lignans, are compounds produced in plants and have estrogenic or anti-estrogenic activities in the human body [8, 9]. Phytoestrogens have the potential to influence various physiological and pathological processes associated with reproduction, bone remodeling, skin, cardiovascular system, nervous system, immune system, and metabolism, and plant-based estrogens are potentially helpful in preventing and treating menopausal disorders [10–12]. It has been reported that phytoestrogens can bind to intracellular estrogen receptors (ER), predominantly found in skin fibroblasts, leading to the activation of intracellular signaling pathways that regulate collagen synthesis [13, 14]. Furthermore, these compounds also demonstrate antioxidative functions and alleviate inflammation, thereby reducing collagen damage [14, 15]. Additionally, phytoestrogens are involved in the suppression of matrix metalloproteinases (MMPs) expression, which contributes to the decreased breakdown of collagen [16]. However, the utilization of phytoestrogens poses potential challenges due to their involvement with numerous targets and both estrogen receptor-dependent and independent mechanisms of action [11]. The disparities observed between findings from experimental and clinical studies and the availability of reliable sources of phytoestrogens have been subjects of discussion. Continuous research on the mechanisms underlying different diseases and their symptoms is imperative, particularly in the increasing prevalence of products incorporating phytoestrogens.

In the previous study, we developed isoflavone-enriched soybean (Glycine max) by preharvest treatment of ethylene [17] and, in this study, we investigated the effects of isoflavone-enriched soybean leaves (IESL) on the loss of collagen following ovariectomy (OVX) in SD rats.

## Main text

### Phytoestrogen-riched soybean leaves extract

**Fig. 1:**
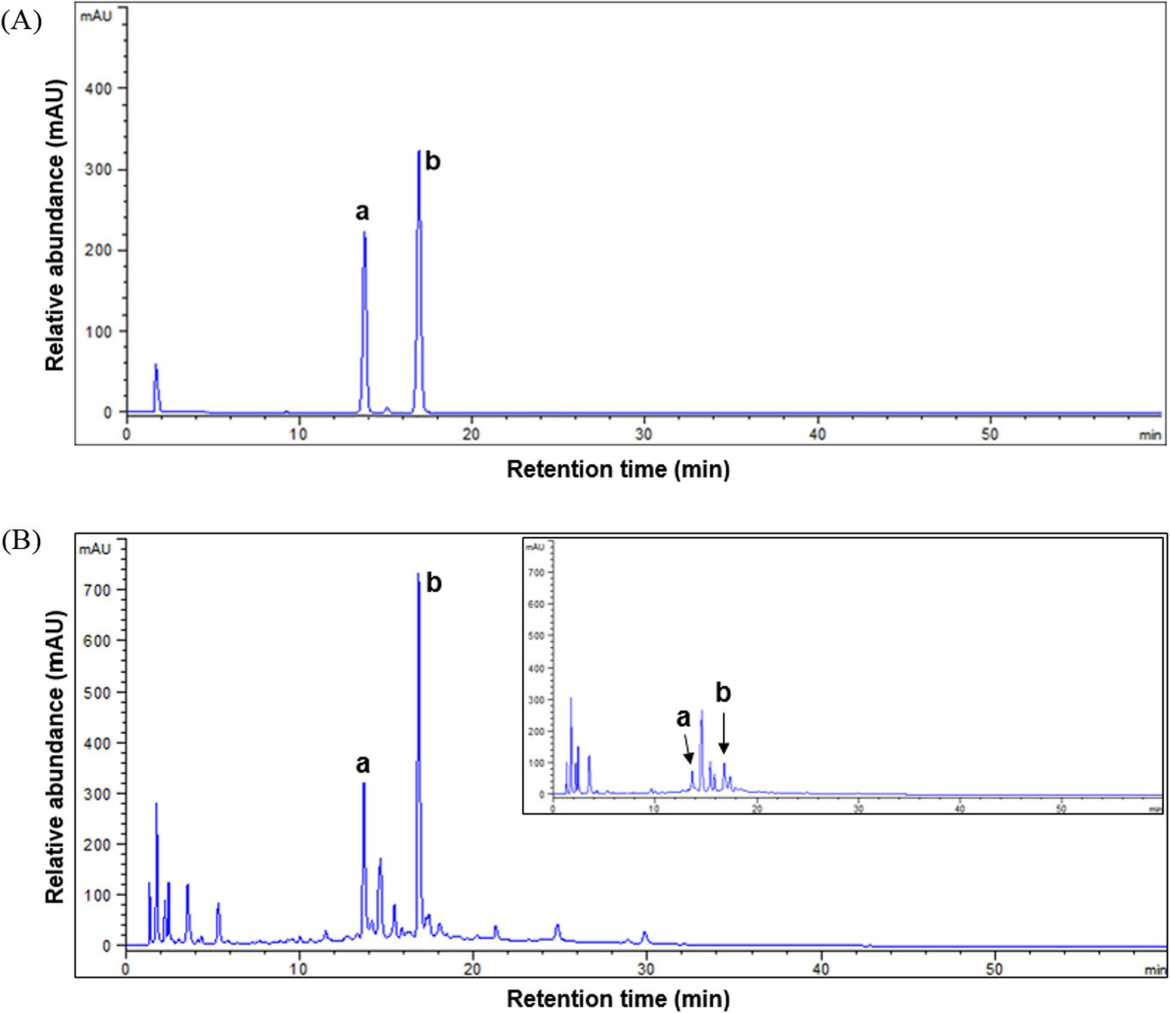
HPLC chromatograms **A** Two isoflavone standards. **B** Isoflavone derivatives in hot-water extract of isoflavone-enriched soybean leaves (IESL), insert: typical soybean leaves. a: daidzin; b: genistin

IESLs were obtained by metabolite farming through ethylene treatment to soybean plants at Gyeongsang National University [17]. Metabolite farming is a particular procedure for preharvest or post-harvest plants to enhance the content of bioactive metabolites. IESL has the feature of having around 50-fold high isoflavone content in comparison with typical soybean leaves (Fig. 1A, B, and insert). Target isoflavones were annotated as daidzin and genistein by LC-ESI-Q-TOF–MS spectral data and comparing retention time (*t*_R_) with standard compounds. IESL extracts were concentrated to have an 11 mg/g content of isoflavones.

### Inducing OVX in SD rats

Female Sprague Dawley (SD) rats, aged six months, were procured from Central Lab Animal Inc. (Seoul, South Korea). Following a seven-day acclimation period, the rats underwent a bilateral ovariectomy. After the surgery, the animals were categorized into four different groups: the first control group consisted of intact rats that were given only a water vehicle (CTL); the second group included ovariectomized rats that received the water vehicle (OVX); the third group was made up of ovariectomized rats received IESL daily containing a lower dosage of isoflavones (OVX/L; 6.25 mg/kg/day of isoflavones); finally, the fourth group involved ovariectomized rats given IESL containing a higher dosage of isoflavones (OVX/H; 18.8 mg/kg/day of isoflavones). Oral administration of IESL was conducted each day for a total duration of three months. Animals were maintained according to the guidelines of NIH for the care and use of laboratory animals. All experimental protocols and surgical procedures were approved by the Institutional Animal Care and Use Committee of Gyeongsang National University (Approval no. GNU-150804-R0037).

### Loss of dermal collagen fibers and roles of IESL

**Fig. 2:**
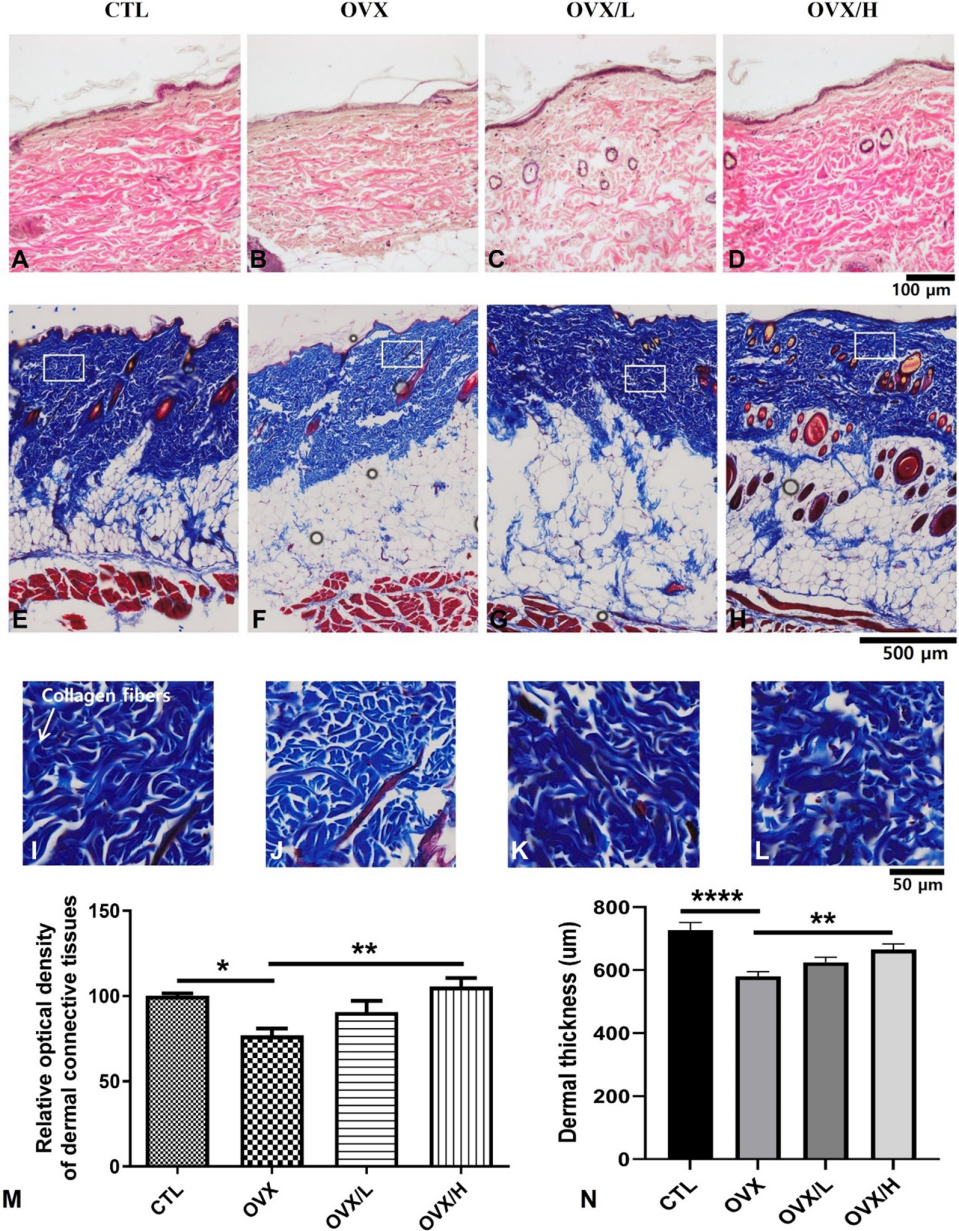
Hematoxylin and Eosin staining (H&E staining, **A**–**D**) in rat skin and Masson's trichrome staining (**E**–**H**) for collagen fibers and magnified views (**I**–**L**) in the CTL (n = 5), OVX (n = 6), OVX/L (n = 9) and OVX/H (n = 9) groups. Relative optical density (ROD, **M**) and thickness (**N**) of dermal connective tissues. Dense collagen fibers are detected in the dermis in the CTL group, but OVX significantly decreased the ROD of the dermal connective tissues. In the OVX/H group, however, treatment of IESL significantly restored the ROD value compared to that of the OVX group. *p < 0.05, **p < 0.01, and ****p < 0.0001

In the CTL group, well-developed dense connective tissues are detected in the dermis both in hematoxylin and eosin (H&E) staining and Masson’s trichrome (MT) staining (Fig. 2A, E). Upon examination of the magnified images from the CTL group, it is evident that the collagen fibers exhibit a remarkably pronounced staining, indicating a strong intensity (Fig. 2I). OVX surgery significantly decreased the relative optical density (ROD) of collagen fibers in the dermis (Fig. B, F, and J), showing a reactive optical density of 76.801% compared to the control group (Fig. 2M). In the OVX/L group, a significant change in the ROD values was not observed compared to the OVX group (Fig. C, G, and K). In the OVX/H group, however, the ROD value increased significantly compared to the OVX group (Fig. D, H, and L), with a value of 105.515% compared to the control group (Fig. 2M). Dermal thickness exhibited a similar pattern of change as the ROD value in the dermis (Fig. 2N). In the control group, the average dermal thickness measured 726.824 μm, while in the OVX group, it decreased significantly to an average of 580.167 μm. The OVX/L group showed increased average values of 90.507% and 625.292 μm for ROD and dermal thickness, respectively, compared to the OVX group, but the significance was not observed when compared to the OVX group. In the OVX/H group, dermal thickness was significantly increased compared to the OVX group, with an average value of 664.75 μm observed.

In this experiment, IESL that we used have about 50 times the isoflavone content compared to regular soy leaves and were confirmed to contain isoflavones such as daidzin and genistein (Fig. 1). Isoflavones, found in plants, are known to exhibit various physiological effects in the human body by mimicking the action of estrogen [18]. Such isoflavones are also known to be involved in skin protection through the up-regulation of antioxidants and their free radical scavenging activities [19, 20]. They are also closely involved in modulating collagen production and reduction in the skin [21, 22]. In the previous study, we confirmed that treatment of IESL significantly increased *COL1A1* and *COL3A1* expression in human dermal fibroblasts [21]. Furthermore, there are numerous research findings indicating that isoflavones influence the production of collagen in the skin.

Concerning TGF-β (Transforming Growth Factor-Beta) and Smad proteins, it has been widely reported that isoflavones activate TGF-β receptors, enhancing the phosphorylation of Smad proteins and increasing collagen production [23, 24]. In addition, menopause induces an imbalance between MMPs and their inhibitors [25], and it can result in excessive collagen degradation and tissue damage, contributing to skin aging [26, 27]. Isoflavones can bind to estrogen receptors and mimic the actions of endogenous estrogens, including modulation of MMPs via the MAPK and AP-1 pathways [28].

In this study, we utilized a specially cultivated IESL, which had approximately 50 times more isoflavones, and notably observed a significant increase in collagen density in the dermis of the ovariectomized rat skin. Given these results, IESL could be developed as an effective therapeutic agent in mitigating skin aging and promoting tissue damage recovery.

## Conclusions

In the present study, we demonstrated the potential of IESL in mitigating collagen fiber loss due to ovariectomy in SD rats, mimicking the menopausal conditions in women. The use of IESL significantly counteracted the reduction in collagen fibers' relative optical density and dermal thickness, indicating the beneficial role of isoflavones. The effects were more pronounced at higher isoflavone dosages, suggesting a dose-dependent response. Our results underscore the potential of IESL as a practical approach to address collagen loss and skin aging associated with menopause.

## Data Availability

The datasets used and/or analyzed during the current study are available from the corresponding author upon reasonable request.

## Abbreviations

IESL: Isoflavone-enriched soybean leaves
CTL: Control
OVX: Ovariectomy
OVX/L: Ovariectomy + low dose
OVX/H: Ovariectomy + high dose
SD rats: Sprague Dawley rats
MMPs: Metalloproteinases
TGF-β: Transforming Growth Factor-Beta
